# Improvements in Image Registration, Segmentation, and Artifact Removal in ThermOcular Imaging System

**DOI:** 10.3390/jimaging11050131

**Published:** 2025-04-23

**Authors:** Navid Shahsavari, Ehsan Zare Bidaki, Alexander Wong, Paul J. Murphy

**Affiliations:** 1 School of Optometry and Vision Science, University of Waterloo, Waterloo, ON N2L 3G1, Canada; navid.shahsavari@uwaterloo.ca (N.S.); paul.murphy@uwaterloo.ca (P.J.M.); 2 System Design Engineering Department, University of Waterloo, Waterloo, ON N2L 3G1, Canada; alexander.wong@uwaterloo.ca

**Keywords:** infrared thermography, ThermOcular, semantic segmentation, tear film analysis

## Abstract

The assessment of ocular surface temperature (OST) plays a pivotal role in the diagnosis and management of various ocular diseases. This paper introduces significant enhancements to the ThermOcular system, initially developed for precise OST measurement using infrared (IR) thermography. These advancements focus on accuracy improvements that reduce user dependency and increase the system’s diagnostic capabilities. A novel addition to the system includes the use of EyeTags, which assist clinicians in selecting control points more easily, thus reducing errors associated with manual selection. Furthermore, the integration of state-of-the-art semantic segmentation models trained on the newest dataset is explored. Among these, the OCRNet-HRNet-w18 model achieved a segmentation accuracy of 96.21% MIOU, highlighting the effectiveness of the improved pipeline. Additionally, the challenge of eliminating eyelashes in IR frames, which cause artifactual measurement errors in OST assessments, is addressed. Through a newly developed method, the influence of eyelashes is eliminated, thereby enhancing the precision of temperature readings. Moreover, an algorithm for blink detection and elimination is implemented, significantly improving upon the basic methods previously utilized. These innovations not only enhance the reliability of OST measurements, but also contribute to the system’s efficiency and diagnostic accuracy, marking a significant step forward in ocular health monitoring and diagnostics.

## 1. Introduction

Infrared (IR) thermography is a valuable non-invasive technique that can detect subtle changes in ocular surface temperature (OST). By capturing detailed thermal profiles, IR thermography helps examine temperature variations across the entire ocular surface or from specific regions of interest (ROI), for example, central cornea. OST measurements are valuable for healthcare clinicians in identifying abnormalities in tear film dynamics, eye inflammation, and early signs of disease, including dry eye disease, glaucoma [1], inflammation of the lacrimal drainage system [2], age-related macular degeneration, and diabetic retinopathy [3,4].

IR thermography systems can be grouped into two categories: single-camera and dual-camera systems, which operate under three control methods: manual, semi-automatic, and automatic. Single-camera systems rely solely on an IR camera, which limits the accurate localization of specific ROI due to the lack of visible landmarks in thermal images. Dual-camera systems, on the other hand, integrate a visible (VIS) camera with the IR camera to enable more precise localization by synchronizing thermal and visual data. This synchronization facilitates accurate corneal delineation in the IR image, which is critical for ocular surface temperature (OST) measurement. While the use of dual-camera systems is not novel, their application in OST measurement provides distinct advantages by addressing the limitations of single-camera setups. Recent studies have also explored alternative approaches to increase accessibility, including smartphone-based infrared thermography for ocular surface imaging [5]. However, dual-camera systems often rely on manual or semi-automated data analysis processes, which introduce challenges in achieving consistent and efficient measurements [6,7].

OST analysis has relied heavily on manual selection methods, in which specific corneal points were chosen for study. Though straightforward, these techniques were susceptible to subjective bias and inconsistency [8,9]. The introduction of semi-automated and automated methods marked a significant advancement, aiming to reduce manual intervention and improve measurement accuracy [10,11]. Semi-automatic methods reduced the manual effort, but still required user input for the initial setup or during the analysis phase [10]. Automated techniques, utilizing advanced image processing algorithms such as active contour models and snake algorithms, further reduced the need for manual input, aiming for autonomous corneal boundary identification and thus, more precise OST assessments [12,13]. Improving the accuracy of OST measurements has important clinical implications, as it can enhance the early detection and monitoring of ocular surface disorders such as dry eye disease, inflammation, and retinal conditions.

**Figure 1:**
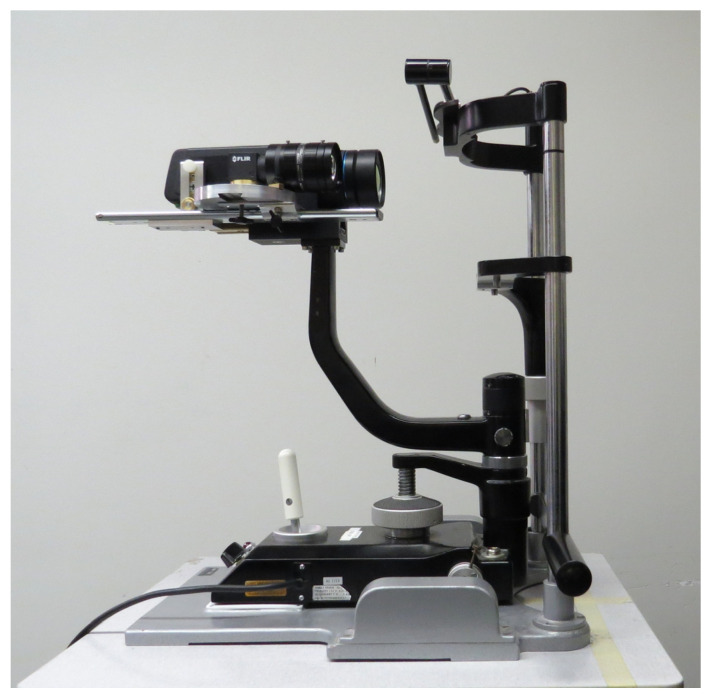
The ThermOcular device used for ocular surface temperature assessment.

ThermOcular is an innovative OST system for comprehensive imaging and segmentation that enhances the precision of OST measurements by accurately tracking the corneal ROI in the IR image (Figure 1). ThermOcular uses a dual-camera setup that captures synchronized IR and VIS video streams to locate and track temperature on the eye surface. After recording synchronized video files of the eye surface, ThermOcular uses custom algorithms to process the video and extract the corneal ROI temperature profile.

For data collection, two synchronized video recordings (IR and VIS) are captured. Next, corresponding control points (CPs) are selected on the first pair of IR and VIS frames. These points are then tracked and localized using an optical flow algorithm. With the CP identified for all frames, the videos are registered. Following registration, a segmentation algorithm is applied to the VIS images to localize the cornea ROI. Once the cornea is localized in the VIS images, the corresponding ROI is mapped onto the IR images, allowing the temperature profile to be extracted [14,15]. Previous work with the ThermOcular system has demonstrated its comparative advantages over existing OST systems, particularly in terms of accurate corneal localization [15]. Building on that foundation, the current study focuses on improving the system’s registration, segmentation, and artifact removal components to further enhance its clinical utility.

Despite these advancements, several challenges remain in achieving fully automated and precise OST measurement. One significant issue lies in the selection of CPs within IR and VIS frames, which can lead to inaccuracies if the CPs are not accurately identified and tracked throughout the video sequence. Errors in optical flow tracking can accumulate over successive frames due to small head or eye movements, noise, or tracking drift. These cumulative inaccuracies, known as optical flow propagation errors, lead to the misalignment of control points, ultimately reducing registration accuracy between the IR and VIS frames. To mitigate this issue, we implemented a static control point assignment approach, selecting CPs only in the initial frame and keeping them fixed throughout the sequence. Additionally, the introduction of EyeTags as stable reference markers further enhances registration precision by reducing reliance on dynamic tracking. Achieving the high-accuracy segmentation of the cornea is essential, as precise delineation directly impacts the reliability of OST measurements. Effective segmentation ensures that the temperature profile is accurately extracted from the corneal ROI, enhancing the clinical relevance of the measurements. Furthermore, the removal of eyelash artifacts and the accurate detection of blinks are critical for maintaining uninterrupted temperature tracking.

Building upon the foundational advancements made with the ThermOcular system for OST assessment, this paper presents a series of enhancements to elevate the system’s measurement precision and clinical utility. A primary improvement introduced in this study was the refinement of CP selection to improve registration accuracy. By designating initial frames with static CPs to register all subsequent frames, the system accounts for small head and eye movements by the subject and reduces errors associated with optical flow. Additionally, EyeTags were integrated as visual markers, aiding clinicians in precise CP selection and minimizing potential human error, thus enhancing registration consistency across frames.

Following these registration improvements, advanced segmentation techniques were employed to enhance the accuracy of corneal ROI isolation. High-accuracy segmentation is crucial for ensuring that temperature readings are localized to the corneal ROI, minimizing any interference from surrounding ocular structures. New semantic segmentation models, trained on an extensive dataset, were developed to enhance the system’s ability to reliably identify ocular components.

This study also addressed the artifacts from the eyelashes and eye closure due to blinking that interfere with temperature readings. The enhanced methodology successfully eliminated these artifacts, while a refined blink detection and removal algorithm ensured continuous and accurate OST measurement.

With these advancements, the ThermOcular system achieves greater diagnostic accuracy and usability, establishing a new benchmark for OST measurement in clinical ocular health applications. This paper reviews the literature underpinning these developments and details the methodology for system enhancements, followed by a comprehensive analysis of results and insights for future research directions.

## 2. Materials and Methods

This section outlines the methodologies employed to enhance the ThermOcular system’s performance in extracting and tracking temperature in different ROI. The advancements focus on three areas of improvement: registration, segmentation, and artifact removal. These enhancements address the limitations observed in the current system.

### 2.1. Improvements in Registration Process

The ThermOcular system uses an optical flow algorithm for dynamic control point updates across successive frames, a method designed to compensate for patient movements and ensure accurate registration. However, this technique, despite its adaptability, introduced errors. This led to a reassessment of the registration strategy to improve system precision and reliability.

#### 2.1.1. Static Control Point Assignment

A new method was introduced for assigning control points (CPs) in the initial frame without updating them in subsequent frames. This static CP approach leverages the minimal head and eye movement typically observed during short imaging sequences to maintain consistent reference points. By eliminating the need for dynamic updates through optical flow, this method reduces the risk of errors that accumulate over time due to inaccuracies in tracking. As a result, it has the potential to significantly enhance registration accuracy, particularly for precise applications like ocular surface temperature (OST) measurement.

#### 2.1.2. Integration of EyeTags

**Figure 2:**
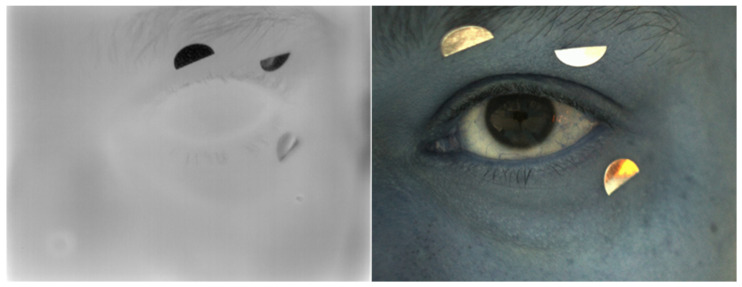
A visual comparison of the EyeTags as seen in IR (**left**) and VIS (**right**) imaging, demonstrating their clear visibility and distinct pattern, which facilitated accurate registration in the ThermOcular system.

To improve registration accuracy, EyeTags were placed on the skin near the inner and outer canthus of the eye. These locations were selected due to their stability (minimal movement relative to the eye) and thermal visibility (clear contrast in both infrared and visible images). Additionally, placing the EyeTags in these regions ensures precise alignment without interfering with corneal temperature measurement, thus maintaining accurate registration across frames (Figure 2). EyeTags enhance the ThermOcular system’s performance by serving as stable, easily identifiable reference points in both IR and VIS images. This approach simplifies manual control point selection, reducing dependency on subjective judgment. By providing well-defined landmarks, EyeTags help maintain high registration precision, significantly minimizing human error and ensuring more accurate alignment between IR and VIS imagery of the ocular surface.

While the placement of EyeTags requires minimal training, the process is designed to be intuitive, reducing the learning curve for clinicians. To further enhance usability and adoption, future work will focus on automating control point selection through deep learning-based landmark detection, reducing reliance on manual placement. Additionally, workflow optimizations, such as guided visual prompts and real-time feedback mechanisms, will be explored to assist clinicians in accurate marker positioning. These enhancements aim to improve system accessibility and ease of use, ensuring higher adoption rates in clinical practice.

#### 2.1.3. Performance Evaluation (Control Point Tracking)

The efficacy of the registration process was evaluated by comparing the use of EyeTags with the manual CP method, employing both static and optical flow methods for CP retention. A single video sequence was captured (of over 500 frames), with CPs manually established on either an eye feature or an EyeTag to assess registration accuracy under different scenarios.

Four scenarios were designed to assess the effectiveness of static and optical flow methods of CP retention, with and without the use of EyeTags. The Root Mean Square Error (RMSE) was calculated for each scenario to quantify the registration error between the predicted CP and a manually selected ground truth across all frames (Table 1).

While optical flow is typically expected to improve registration by adjusting to small object movements, its effectiveness is highly dependent on the accuracy of feature tracking across frames. As shown in Table 1 and Table 2, when no stable reference points were present, optical flow exhibited increased RMSE and FRE values, indicating significant misalignment over time. This is primarily due to tracking drift and accumulated errors in motion estimation, which led to a decline in registration accuracy rather than improvement.

However, the inclusion of EyeTags as stable reference markers significantly mitigated these tracking errors, providing fixed points for alignment. The results indicate that optical flow, when paired with EyeTags, achieved the lowest RMSE values in VIS frames, demonstrating that robust reference points are essential for minimizing registration errors. These findings highlight the need for structured control points in small-region imaging, particularly in ocular surface temperature (OST) measurement, where even minor misalignments can lead to unreliable results.

#### 2.1.4. Fiducial Registration Error Calculation

To further assess the precision of the registration process in the ThermOcular system, the fiducial registration error (FRE) was calculated. The FRE is a metric used to quantify the misplacement of CP during registration, representing the overall misalignment of points. The Euclidean distance between the reference points in the fixed frame (VIS) and the corresponding points in the transformed image (IR) was determined. The FRE was computed as the average of these Euclidean distances.

Eight CP were identified in both the fixed and moving frames. A transformation matrix was then calculated using three pairs of corresponding points. The remaining CPs were transformed using this matrix and the distances between the transformed points and their original counterparts were computed. The mean of these distances for each frame was taken as the FRE for that frame. This process was applied to all frames in the sequence and the resulting FREs were averaged to yield the mean FRE for each registration scenario. The results are presented in Table 2.

### 2.2. Improvements in Segmentation Process

The segmentation process was refined by evaluating the performance of several state-of-the-art semantic segmentation models on the Apricot dataset of ThermOcular IR and VIS video sequences from research in our lab.

#### 2.2.1. Dataset Preparation

The Apricot dataset comprises machine vision videos of 84 subjects, ensuring a diverse representation of eye color, fixation patterns, and eye aperture openness. Participants were recruited based on the following inclusion criteria: healthy individuals aged 18–45 with no history of ocular disease, no recent eye surgery, and no use of topical ocular medications. Exclusion criteria included contact lens wear, systemic conditions affecting tear film stability, and recent ocular infections. These criteria ensured that the ocular surface temperature (OST) measurements were not influenced by external medical conditions that could alter thermal readings. To ensure consistency in ocular surface temperature (OST) measurements, all imaging sessions were conducted in a temperature-controlled room (22 °C ± 1 °C), with the relative humidity maintained at 40–50%. Before recording, each participant underwent a 5 min adaptation period to allow for tear film stabilization, minimizing external factors affecting thermal measurements.

Through careful selection, 15 images from each subject were extracted to cover a spectrum of eye conditions. Augmentation was further enriched by integrating 2000 images from the TEyeD dataset [16] to address any imbalance with replication and to maximize the dataset’s diversity. For segmentation model training, we used an 80/20 train/test split. Cross-validation was not applied in this study but is planned for future work to enhance model generalizability and reproducibility.

#### 2.2.2. Model Selection and Training Details

To ensure the best segmentation results, several semantic segmentation models were evaluated, including PSPNet [17], Deeplabv3 [18], PP-LiteSeg [19], Unet++ [20], SegFormer [21], and OCRNet [22]. These models were chosen due to their proven effectiveness in various semantic segmentation challenges, particularly in medical image segmentation tasks. The optimal configuration used a batch size of five over 15,000 iterations, with a learning rate of 0.01. The model was trained with the SGD optimizer and the cross-entropy loss function. HRNet-w18 was used as the backbone, pre-trained on the VOC12 dataset and then fine-tuned on the Apricot dataset.

#### 2.2.3. Performance Evaluation (Segmentation)

To assess the effectiveness of segmentation, we evaluated multiple state-of-the-art semantic segmentation models, each pre-trained on publicly available datasets optimized for general segmentation tasks. The models were then fine-tuned on our dataset to measure their performance in ocular surface segmentation. Different models were originally trained on different datasets (e.g., ImageNet, VOC12, Cityscapes) because they were designed for varying segmentation tasks. Using their respective pre-trained versions allows us to compare how well each model generalizes to our specific problem domain after fine-tuning, rather than assessing their original pre-training performance. This approach provides a more realistic comparison of their adaptability to OST segmentation. Table 3 summarizes the segmentation performance of the models based on the Mean Intersection Over Union (MIOU) metric, highlighting the accuracy differences across architectures.

### 2.3. Eyelash Elimination

A notable limitation in the existing ThermOcular system’s segmentation was the inability to exclude eyelashes from the IR images. The presence of eyelashes, with their distinct temperature, skews the temperature readings of the cornea ROI, leading to imprecise outcomes. In addressing this issue, this study used a method to eliminate the influence of eyelashes on the IR image. Since eyelashes have a temperature significantly cooler than the ocular surface, they can be identified as outliers within the cornea and sclera ROIs.

Assuming a normal temperature distribution across the ROI, the eyelash temperature deviates considerably from the mean. This assumption is based on empirical observations of temperature profiles in healthy eyes. Future work will include formal statistical tests to validate the normality of temperature distributions across diverse clinical populations. To pinpoint and eliminate these outliers, the following outlier detection strategy was employed:

(1) $$\mu =\mathrm{mean}\;\left(\mathrm{ROI}\_\mathrm{temperature}\right)$$

(2) $$\sigma =\mathrm{std}\;\mathrm{dev}\;\left(\mathrm{ROI}\_\mathrm{temperature}\right)$$

(3) Lower limit=μ−2σ

(4) Upper limit=μ+2σ

Pixels falling outside the bounds of the Lower Limit and Upper Limit (either warmer or cooler by more than two standard deviations from the mean, respectively) are flagged as outliers and removed from the IR image. Empirical results confirmed the efficacy of this approach, showcasing IR images devoid of eyelash interference. This refinement ensured a more accurate representation of the true temperature properties of the cornea ROI, enhancing the reliability of the ThermOcular system.

**Figure 3:**
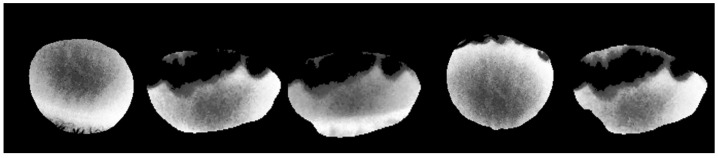
Sample IR frames of the cornea ROI from five different eyes, demonstrating the effectiveness of the eyelash removal method. The black areas represent the removed eyelashes, ensuring that only the corneal surface is analyzed for temperature measurement. In these IR images, darker areas correspond to cooler regions, while brighter areas indicate warmer regions. The ocular surface temperature typically ranges between 32 °C and 36 °C, depending on environmental conditions and physiological factors.

To evaluate the performance of our eyelash removal method, we applied it to IR images from five different corneas, as shown in Figure 3. The variation in the outer contours is due to differences in individual eye shapes. The primary purpose of this figure is to showcase the successful removal of eyelashes, which appear as black regions, rather than to compare corneal shapes. For better interpretability, we note that in these infrared images, darker areas indicate cooler temperatures, while brighter areas correspond to warmer regions.

### 2.4. Blink Elimination

A significant challenge for the IR imaging of the eye is the presence of an eyelid blink in a video sequence. The ThermOcular system addressed this by eliminating frames where no cornea is detected. However, this method was susceptible to inaccuracies, particularly in instances where the eye was not fully closed, compromising the reliability of the captured data.

**Figure 4:**
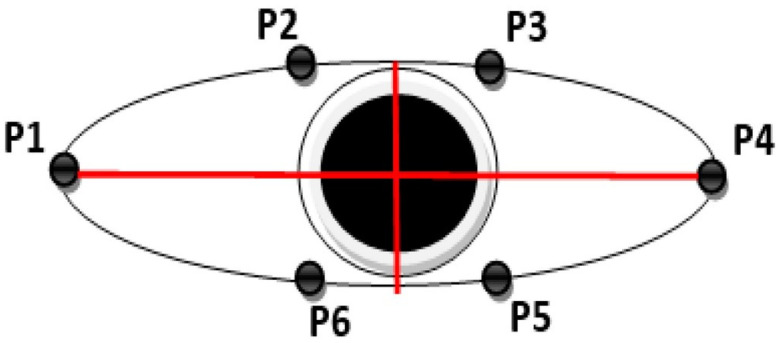
Eye Aspect Ratio (EAR) method for blink detection, illustrating the anatomical landmarks used for calculating the EAR during a blink sequence.

To enhance the accuracy of blink detection, this study used the Eye Aspect Ratio (EAR) as an alternative metric. The EAR provides a consistent estimate of the eye’s openness by measuring the ratio of distances between the vertical landmarks and the horizontal landmarks of the eye (Figure 4) [23].

(5) $$EAR=\frac{\left|\left|P2-P6\right|\left|\;+\;\right|\left|P3-P5\right|\right|\;}{2\times \left|\left|P1-P4\right|\right|}$$

where *P*1, *P*2 …, *P*6 are the landmarks on the eye.

Frames where the EAR falls below a threshold of 0.3 were flagged as blinks and excluded. This approach provides a more nuanced understanding of eye closures. It effectively distinguishes between complete blinks and situations where the eye is only partially closed, ensuring that the IR data are accurate and reliable.

## 3. Conclusions

**Figure 5:**
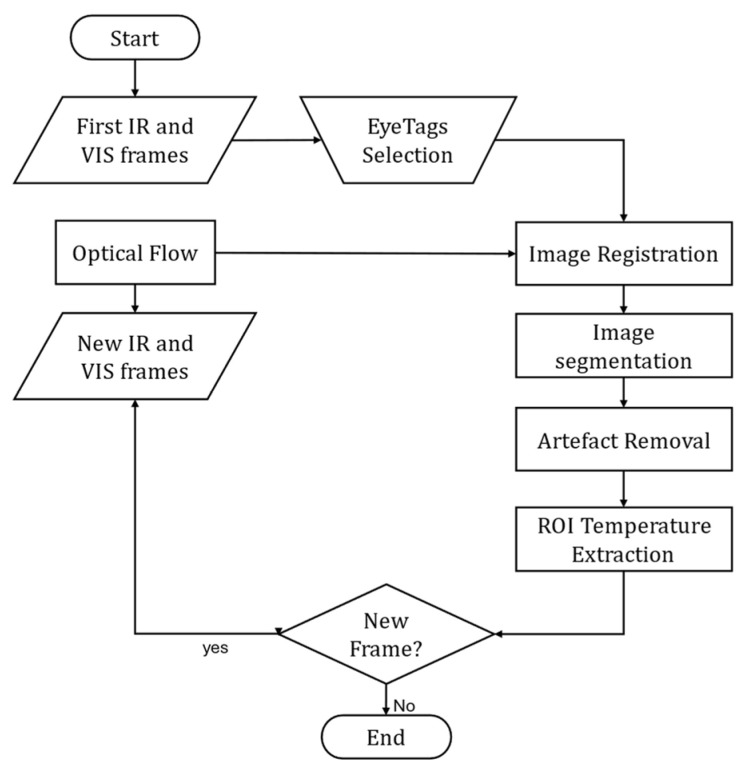
Revised ThermOcular system algorithm flowchart for video registration (incorporating EyeTags) and image segmentation for region of interest (ROI), including artifact removal.

In this paper, improvements to the ThermOcular system were presented, which enhance its utility for assessing OST. The segmentation process has been substantially improved, allowing for a more accurate identification of key ocular ROI, such as the cornea and sclera. This refinement permits precise temperature readings across the ocular surface, which is crucial for diagnosing a wide range of ocular conditions. Moreover, robust methods were developed for removing artifacts, such as eyelashes and blinks, from the IR images, thereby improving the reliability of OST measurement. The registration process was also optimized by the use of EyeTags to simplify clinician use, reducing the need for extensive training on CP selection and minimizing errors in manual input. Figure 5 shows the revised algorithm flowchart of the new ThermOcular System as result of the conclusions in this study.

While EyeTags improved registration precision and simplified the clinician workflow, their impact on patient comfort has not been formally assessed. The materials used in this study were not designed specifically for medical or prolonged skin contact. As such, future work will involve evaluating user experience and exploring alternative marker materials that are clinically approved, hypoallergenic, and optimized for comfort and safety in diverse patient populations.

While the advancements described have markedly improved the capabilities of the ThermOcular system, several areas remain for future research and development. Firstly, further work is needed to fully automate the detection of ocular landmarks, thereby reducing or eliminating the need for manual CP selection. Such automation would enhance the system’s usability and make it more autonomous. Secondly, future improvements could involve integrating an embedded GPU-based computational infrastructure into the device, addressing the limitations of outdated clinical workstations and enabling the execution of deep learning models directly on the device. This would facilitate faster, real-time analysis while maintaining compliance with healthcare privacy standards by processing data locally. Thirdly, while improvements in segmentation accuracy have been demonstrated, potential segmentation errors remain due to variations in lighting conditions, eye shape, or occlusions (e.g., excessive eyelashes). Moreover, future improvements will aim to enhance segmentation robustness by accounting for inter-individual anatomical variability, such as differences in eyelid anatomy, eye shape, and eyelash density. Future efforts should focus on improving the robustness of segmentation models by expanding the training dataset and refining algorithms to handle diverse cases more effectively. Fourthly, the generalization of the ThermOcular system to different populations is a challenge, as the dataset used in this study primarily consists of healthy individuals. Future clinical validation will involve a broader population, including patients with ocular conditions such as dry eye disease, ocular inflammation, diabetic retinopathy, and corneal injuries, to evaluate the system’s diagnostic utility and robustness in real-world clinical scenarios. Finally, the computational requirements of high-performance segmentation models, particularly those with HRNet backbones, may pose challenges for real-time clinical deployment. Future research should focus on optimizing these models for embedded GPUs or edge computing, enabling more efficient and accessible clinical use.

Despite these limitations, the improvements introduced in this study provide a solid foundation for advancing ocular thermography as a reliable and non-invasive diagnostic tool. Future research will address these challenges by refining segmentation techniques, expanding the dataset to include a more diverse population, and optimizing computational efficiency for real-world clinical applications, including deployment on embedded GPUs and edge computing platforms to accommodate resource-limited settings.

Notably, the OCRNet-HRNet-w18 segmentation model achieved a mean Intersection Over Union (MIOU) of 96.21%, reflecting the effectiveness of the proposed improvements in ocular region detection and overall system accuracy.

## Figures and Tables

**Table 1:** Error calculation results for control point (CP) following methods, with and without EyeTags, in the infrared (IR) and visible (VIS) video frames.

Strategy	CP Following Method	Frame Type	RMSE (in Pixels)
Without EyeTags	Static	IR	10.36
		VIS	9.27
	Optical Flow	IR	13.09
		VIS	11.81
With EyeTags	Static	IR	9.54
		VIS	9.33
	Optical Flow	IR	6.28
		VIS	4.39

**Table 2:** Calculated fiducial registration error (FRE) for each control point following each registration method.

Strategy	CP Assignment Method	Mean FRE ± SD (in Pixels)
Without EyeTags	Static	11.04
	Optical flow	13.36
With EyeTags	Static	9.84
	Optical flow	5.63

**Table 3:** Benchmarking results for semantic segmentation models.

Model-Backbone	Pre-Trained Dataset	MIOU (%)
PSPNet-ResNet50	ImageNet	92.79
Deeplabv3-ResNet50	VOC12	94.23
PP-LiteSeg-STDC2	Cityscapes	91.46
Unet	Cityscapes	91.93
Unet++	Cityscapes	95.63
SegFormer	Cityscapes	94.32
OCRNet-HRNet-w18	VOC12	96.21

## Data Availability

The original contributions presented in this study are included in the article. Further inquiries can be directed to the corresponding author.
